# Behaviors and Mechanisms of Adsorption of MB and Cr(VI) by Geopolymer Microspheres under Single and Binary Systems

**DOI:** 10.3390/molecules29071560

**Published:** 2024-03-30

**Authors:** Yi Fang, Lang Yang, Feng Rao, Kaiming Zhang, Zhuolin Qin, Zhenguo Song, Zhihui Na

**Affiliations:** 1 Zijin School of Geology and Mining, Fuzhou University, Fuzhou 350108, China; fangyi_edu@163.com (Y.F.); 826984234@gmail.com (K.Z.); qzl18378569155@163.com (Z.Q.); 2 Fujian Key Laboratory of Green Extraction and High-Value Utilization of New Energy Metals, Fuzhou 350108, China; 3 State Key Laboratory of Mineral Processing, Beijing 102628, China; 4 Yunnan Phosphate Haikou Co., Ltd., Kunming 650114, China

**Keywords:** geopolymer microspheres, adsorption capacity, competitive adsorption, mechanism

## Abstract

Geopolymers show great potential in complex wastewater treatment to improve water quality. In this work, general geopolymers, porous geopolymers and geopolymer microspheres were prepared by the suspension curing method using three solid waste products, coal gangue, fly ash and blast furnace slag. The microstructure, morphology and surface functional groups of the geopolymers were studied by SEM, XRD, XRF, MIP, FTIR and XPS. It was found that the geopolymers possess good adsorption capacities for both organic and inorganic pollutants. With methylene blue and potassium dichromate as the representative pollutants, in order to obtain the best removal rate, the effects of the adsorbent type, dosage of adsorbent, concentration of methylene blue and potassium dichromate and pH on the adsorption process were studied in detail. The results showed that the adsorption efficiency of the geopolymers for methylene blue and potassium dichromate was in the order of general geopolymers < porous geopolymers < geopolymer microspheres, and the removal rates were up to 94.56% and 79.46%, respectively. Additionally, the competitive adsorption of methylene blue and potassium dichromate in a binary system was also studied. The mechanism study showed that the adsorption of methylene blue was mainly through pore diffusion, hydrogen bond formation and electrostatic adsorption, and the adsorption of potassium dichromate was mainly through pore diffusion and redox reaction. These findings demonstrate the potential of geopolymer microspheres in adsorbing organic and inorganic pollutants, and, through five cycles of experiments, it is demonstrated that MGP exhibits excellent recyclability.

## 1. Introduction

Water pollution has a vast impact on the world. According to statistics, more than 8 million tons of plastic are discharged into the ocean every year, causing damage to marine ecosystems, and about 1 million people die every year from drinking from contaminated water sources [1,2]. In addition, water pollution costs the global economy billions of USD every year. Every year, over 36 billion tons of printing and dyeing wastewater is discharged in China [3]. If this wastewater is not properly treated, the surrounding environment will suffer severe consequences [4]. Methylene blue (MB), a cationic dye commonly used in printing and dyeing and widely discharged into the environment, may cause tissue necrosis, nerve damage and other diseases when it is ingested by humans [5,6]. What is worse, the heavy metal Cr(VI) is found usually co-existing with MB in printing and dyeing wastewater, which may cause diarrhea and kidney damage after excessive exposure [7]. For MB, the treatment methods include adsorption [8,9], membrane separation and photocatalytic oxidation [10,11]. For Cr(VI), adsorption [12,13], chemical coagulation, ion exchange and photocatalysis [14,15] are the mainstream methods. Thus, the common methods to treat both MB and Cr(VI) are adsorption and photocatalytic oxidation, but the utilization rate of sunlight in photocatalytic oxidation is too low at present, and the applied conditions are limited. In other words, adsorption has become the better way to treat MB and Cr(VI) simultaneously because of its simple operation and its being green and environmentally friendly. However, the cost of most adsorbents always limits their applications; it is urgent to develop low-cost and efficient adsorbents [16], such as low-cost activated carbon [17,18,19], natural clay, zeolite, biomass and geopolymers [20,21,22,23,24,25,26].

More and more scholars are becoming interested in geopolymers due to their wide range of cheap raw materials, simple and normal-temperature synthesis process and great adaptive and adsorption capacities [27,28,29,30,31]. The raw materials for geopolymerization reactions typically consist of aluminosilicates and silicoaluminates [32]. Common examples include coal gangue, kaolin, fly ash, slag and tailings. Many industrial waste residues containing active silicon and aluminum can also serve as raw materials for geopolymerization reactions. Under the action of alkaline activators, geopolymer chains can undergo cleavage of -Al-O- bonds and -Si-O- bonds, forming monomeric structures of silicon–oxygen tetrahedra and aluminum–oxygen tetrahedra. These monomeric structures are combined through sharing oxygen atoms to condense into oligomers of aluminosilicate. These oligomers, by altering their silicon-to-aluminum ratio, form geopolymer products that can be classified into four types: (1) When Si:Al = 1, forming (-Si-O-Al) chains, it is known as polysialate geopolymer (PS type). At this point, the geopolymer exhibits a crystalline-like structure resembling a “cage”. (2) When Si:Al = 2, forming (-Si-O-Al-O-Si-) chains, it is termed a polysialate-siloxo geopolymer (PSS type). (3) When Si:Al = 3, forming (-Si-O-Al-O-Si-O-Si-O-) chains, it is referred to as a disiloxo-disialate geopolymer (PSDS type). (4) When Si:Al > 3, it forms polymerized polysialate-siloxo geopolymer chains. These classifications are based on the different ratios of silicon to aluminum in the geopolymer structure, leading to variations in the arrangement of the constituent units and the resulting properties of the geopolymer. Feng et al. [33] prepared a general geopolymer (GGP) with metakaolin (MK) and blast furnace slag (BFS), and they found the adsorption capacity of the geopolymer for MB reached 4.91 mg/g, which proved the geopolymer had great potential for treating printing and dyeing wastewater. Meanwhile, Salma’s [34] experimental results also demonstrated that geopolymers can adsorb MB well, and the adsorption effect was higher than that of Feng’s study. In order to obtain a better adsorption effect, Li [35] used fly ash (FA) as a raw material to prepare a porous geopolymer (PGP) with a certain pore structure that could adsorb MB by acid treatment on the basis of GGPs. Gonçalves [36] was also interested in the adsorption of MB by porous geopolymers; the red mud (RM)-based PGP prepared by 3D printing showed an adsorption capacity of 19.96 mg/g for MB. In order to take into account both the adsorption effect and secondary utilization capacity, Novais [37] proposed the concept of FA-based geopolymer microspheres (MGPs), with an adsorption capacity of MB up to 30.1 mg/g. Bulk-type adsorbents are a safer and easier alternative to powdered adsorbents, and they can be regenerated and reused in multiple adsorption cycles. Although there is considerable research about geopolymers in relation to MB adsorption, there are few studies focusing on their adsorption of Cr(VI). Zhu et al. [38] pointed out that Cr(VI) can adsorb on lignin geopolymers and effectively obtain Cr(VI) adsorption when Fe nanoparticles are added. At the same time, their results showed that the hydroxyl group in lignin geopolymers participates in the reduction process of Cr(VI) to Cr(III). In addition, Yu et al. [39] found the MK geopolymer modified by quaternary ammonium salt could not only adsorb Cr(VI) but also Cu(II) simultaneously. However, the above studies only explore the adsorption performance in a simple organic or inorganic system, never considering the frequent co-existence of organic and inorganic pollutants, e.g., the co-existence of MB and Cr(VI), in dyeing wastewater. Fortunately, previous investigations reminded us of the great adsorption potential of geopolymers for both organic and inorganic pollutants. Thus, it was highly meaningful to study the adsorption behaviors and universal rules of geopolymers in a mixed system. More importantly, the reciprocal effects and mechanisms in the complex adsorption process needed to be explored.

Herein, a new kind of geopolymer microsphere adsorbent (MGP) was prepared by the suspension curing method using industrial solid wastes such as coal gangue (CG), FA and BFS as raw materials. The ratios of raw materials, the modulus of alkali activator and the temperature of the water bath were reasonably designed. Through batch adsorption experiments, the adsorption effect of MB and Cr(VI) was satisfactory. From the perspective of practical application, conditions including the type of adsorbent, adsorbent dosage, initial concentration of MB and Cr(VI) and pH were systematically studied. Moreover, the adsorption mechanism of MGPs for MB and Cr(VI) was confirmed through fitting adsorption kinetics, adsorption isotherm fitting, FTIR and XPS spectrum analysis before and after adsorption. This work provides insights into effective co-adsorption of organic and inorganic pollutants in a binary system with new low-cost geopolymer microsphere adsorbents.

## 2. Results and Discussion

### 2.1. Structures and Morphologies of the Geopolymers

**Figure 1:**
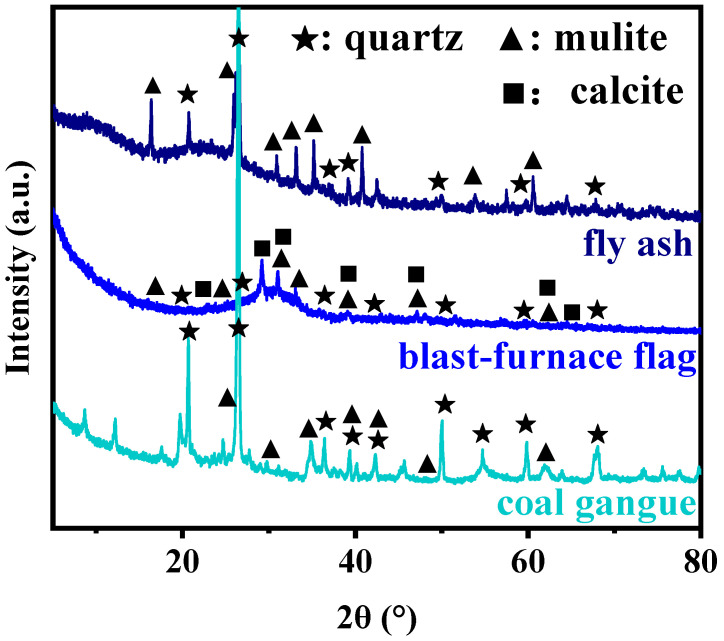
XRD patterns of FA, BFS and CG.

The chemical components of CG, FA and BFS determined by X-ray fluorescence (XRF) are listed in Table 1. The main components of CG and FA are SiO_2_ and Al_2_O_3_, while BFS primarily contains SiO_2_, CaO and Al_2_O_3_. Figure 1 shows the mineralogical characteristics of CG, FA and BFS ascertained through X-ray diffraction (XRD). FA and CG primarily consist of quartz and mullite, whereas BFS also contains the calcite phase.

**Figure 2:**
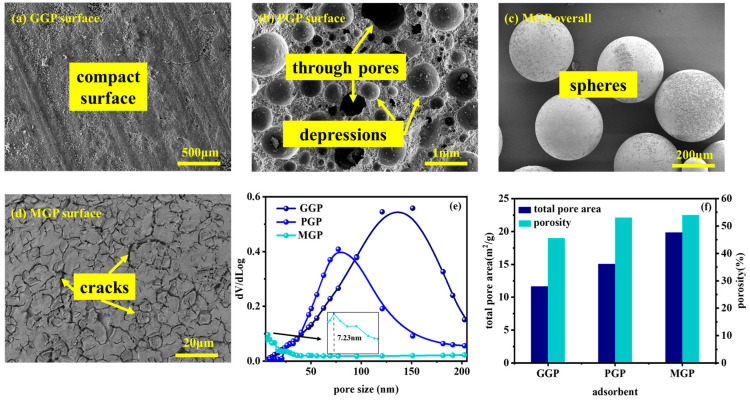
SEM image of (**a**) GGP surface, (**b**) PGP surface, (**c**) MGP overall, (**d**) MGP surface, (**e**) pore size and (**f**) total pore area and porosity of GGP, PGP and MGP.

Figure 2 illustrates the structures, morphologies and pore size distribution of the geopolymers. As shown in Figure 2a, the GGPs typically exhibited a uniform and dense appearance. They possessed a highly crystalline structure with a smooth surface and no significant pores or voids. The morphological features of this type of geopolymer manifested as a consistent and uniform appearance, often used in applications requiring high strength and abrasion resistance, such as concrete repair materials and construction materials. In contrast, after foaming, PGPs exhibited an open or closed pore structure (Figure 2b), presenting a porous and irregular surface. These pores formed a highly interconnected network, providing a large specific surface area and pore volume. Porous geopolymers were commonly used in areas such as adsorbent materials, catalyst supports and thermal insulation materials, exhibiting loose structures and a porous surface. As can be seen from Figure 2c,d, MGPs exhibited a perfect spherical shape with a large number of gullies and cracks on the surface, which provided a large number of adsorption sites and had the advantage of easy recovery. According to Figure 2e, the MGPs were primarily mesoporous materials with pores of approximately 7.23 nm, while the GGPs and PGPs primarily had macropores larger than 50 nm. Mesopores can improve the adsorption efficiency. Regarding total pore area and porosity (Figure 2f), the MGPs could reach 19.81 m^2^/g and 53.96%, respectively. The PGPs were slightly lower than the MGPs, and the GGPs were the lowest.

### 2.2. Factors on the Adsorption of Geopolymers for Methylene Blue and Cr(VI)

**Figure 3:**
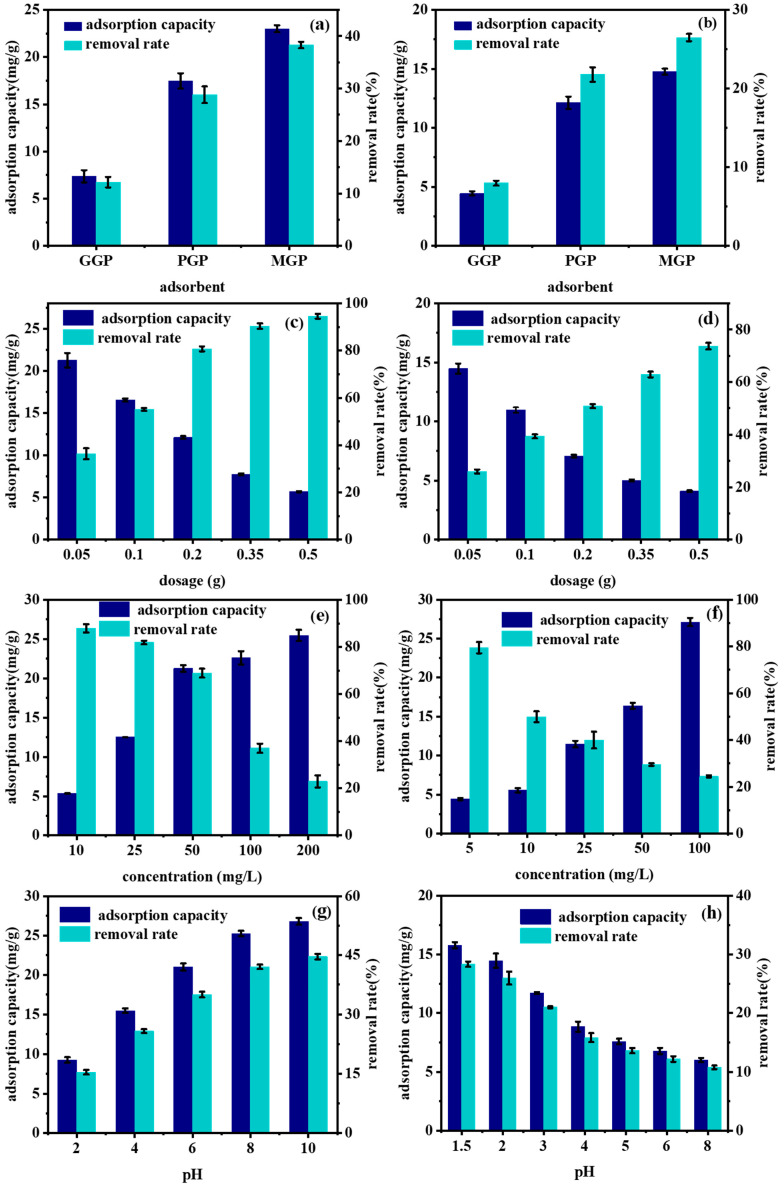
(**a**) MB and (**b**) Cr(VI) adsorption on GGP, PGP and MGP, effect of (**c**,**d**) MGP dosage, (**e**,**f**) initial concentration of adsorbate and (**g**,**h**) pH on MGP for MB and Cr(VI) adsorption.

Figure 3a,b show the adsorption of MB and Cr(VI) on samples obtained through the three preparation methods under the same conditions. It can be seen that GGPs had the lowest adsorption effect, with the adsorption capacity of MB and Cr(VI) reaching only 7.37 mg/g and 4.44 mg/g, respectively. The adsorption effects of the PGPs and MGPs were much higher than that of the GGPs, while the adsorption effect of the MGPs was slightly higher than that of PGPs, reaching 23.01 mg/g and 14.76 mg/g, respectively. The reasons for this are the enlarged specific surface area and porosity, more adsorption sites and adhesion areas and the subsequent boost in adsorption capacity and removal rate of the PGPs and MGPs. Given the MGPs’ superior adsorption capacity, ease of recovery and shorter preparation time, subsequent adsorption experiments were limited to MGPs only.

As shown in Figure 3c,d, with the increasing dosage of adsorbents, the adsorption capacity of the MGPs for MB and Cr(VI) decreased from 21.26 mg/g and 14.47 mg/g to 5.68 mg/g and 4.11 mg/g, respectively, while the removal rates increased from 36.37% and 25.97% to 94.56% and 73.72%, respectively. The enhanced adsorption sites led to better adsorption. With a constant number of adsorbent molecules, most adsorbed onto available sites; however, sites remained unsaturated. This resulted in increased removal rates and decreased adsorption capacity.

As shown in Figure 3e,f, as the initial concentrations of adsorbates increased, the adsorption capacity of MB and Cr(VI) by the MGPs rose from 5.35 mg/g and 4.43 mg/g to 25.46 mg/g and 27.12 mg/g, respectively. However, the removal rates decreased from 87.91% and 79.46% to 22.91% and 24.41%, respectively. This occurred because, as the adsorbate concentration rose, the number of adsorbent molecules per unit volume increased, which gradually saturated the adsorption sites and increased the amount of adsorbed material. At the same time, it became increasingly difficult to remove the adsorbate molecules, leading to a decrease in the removal rate.

As shown in Figure 3g,h, as the pH increased, the adsorption capacity of the MGPs for MB rose from 9.24 mg/g to 26.8 mg/g, and the removal rate increased from 15.38% to 44.63%. The competition between MB molecules and H^+^ diminished with the increasing pH. Additionally, the deprotonation of -OH groups in the MGPs led to increased electrostatic repulsion between MGP structures. This expansion of the geopolymer network structure promoted adsorption [40,41]. Conversely, the adsorption of Cr(VI) by the MGPs decreased with rising pH. This was due to the higher solubility of Cr(VI) at higher pH levels, resulting in more Cr(VI) existing in its oxidized forms (CrO_4_^2−^ and HCrO_4_^−^) in the solution rather than in its more easily adsorbed, reduced form (Cr(III)). Changes in surface charge under high pH conditions altered the charge properties of the adsorbent surface, affecting the adsorption capacity of Cr(VI). Additionally, at high pH levels, other anions such as hydroxide ions (OH^−^) may have competed with Cr(VI) for adsorption onto the solid surface, reducing the efficiency of Cr(VI) adsorption.

### 2.3. Adsorption Kinetics of FA-Based Geopolymer Microspheres for Methylene Blue and Cr(VI)

**Figure 4:**
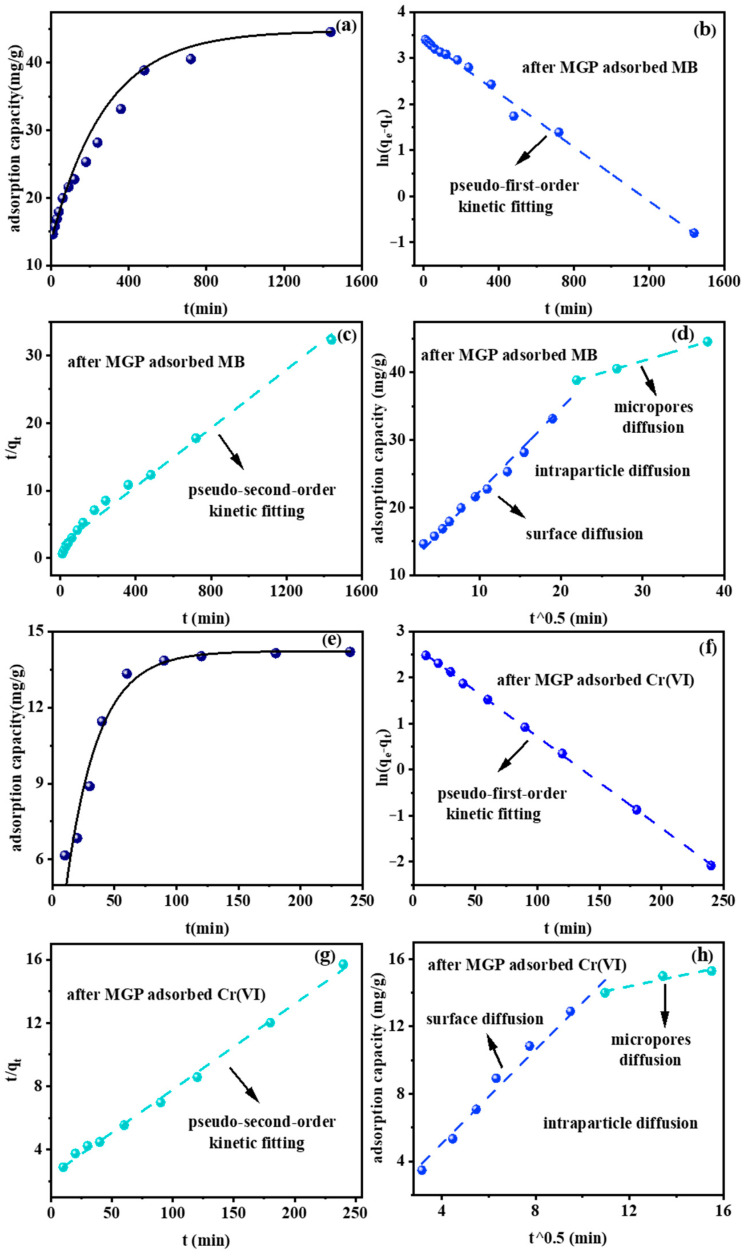
Effect of time and pseudo-first-order, pseudo-second-order and intraparticle diffusion (**a**–**d**) for MB and (**e**–**h**) for Cr(VI).

To further investigate the behaviors of the MGPs in adsorption of MB and Cr(VI), adsorption kinetics fitting was carried out. The linear fitting formulas for the pseudo-first-order kinetic model, pseudo-second-order kinetic model and intraparticle diffusion model are given in Equations (1), (2) and (3), respectively. The fitting parameters are shown in Table 2. Figure 4a,e indicate that the MGPs’ adsorption of MB and Cr(VI) basically reached saturation at around 720 min and 120 min, respectively, and pseudo-first-order kinetic fitting and pseudo-second-order kinetic fitting were performed (Figure 4b,c,f,g). The correlation coefficients R^2^ of the quasi-first-order kinetic fitting and pseudo-second-order kinetic fitting for the MGPs’ adsorption of MB were both equal to 0.99, but the *q_e_* of the pseudo-second-order kinetic fitting shown in Table 2 was similar to the experimental results so the adsorption of MB by the MGPs was more in line with the pseudo-second-order kinetic fitting [42]. The correlation coefficient R^2^ of the MGPs’ pseudo-first-order kinetic fitting for Cr(VI) adsorption was greater than that of pseudo-second-order kinetic fitting so the adsorption of Cr(VI) by the MGPs was more consistent with pseudo-first-order kinetics. The intraparticle diffusion model is shown in Figure 4d,h; this can explain the adsorption behaviors well. The adsorption process of MB and Cr(VI) can be divided into two stages; the first stage is the diffusion of MB from the solution to the surface of the MGPs; the second stage is the diffusion of MB from the surface of the microspheres through the micropores into the interior. The *p* value is not 0, the in-particle diffusion is not a rate control step for the adsorption of MB by microspheres and the actual adsorption rate is jointly controlled by several processes.

(1) $$\ln\left(q_{e}-q_{t}\right)=\ln q_{e}-k_{1}t$$

(2) $$\frac{t}{q_{t}}=\frac{1}{k_{2}{q_{e}}^{2}}+\frac{t}{q_{e}}$$

(3) $$q_{t}=k_{i}t^{0.5}+P$$

where *q_e_* (mg/g) and *q_t_* (mg/g) are the amounts of MB or Cr(VI) adsorbed by the adsorbent at the equilibrium time and the given time; *k*_1_ (1/min) and *k*_2_ (g/mg/min) are the rate constants of the corresponding model; *k_i_* (mg/g/min^0.5^) is the internal diffusion rate constant; *P* is the coefficient related to the thickness of the boundary layer.

### 2.4. Adsorption Isotherms of FA-Based Geopolymer Microspheres for Methylene Blue and Cr(VI)

**Figure 5:**
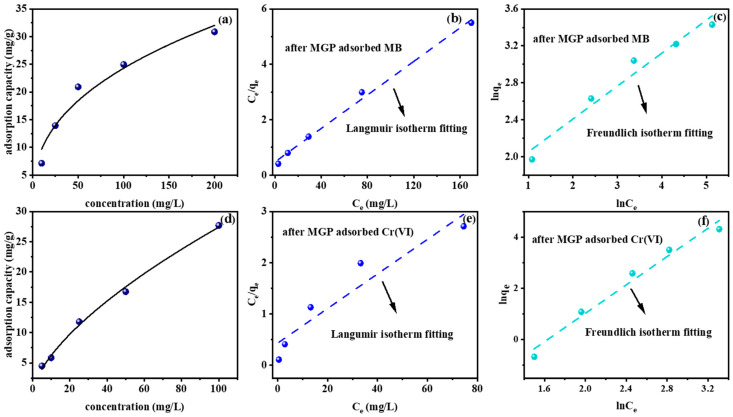
Effect of concentration on (**a**) MB and (**d**) Cr(VI) and Langmuir and Freundlich isotherm fitting of the adsorption of (**b**,**e**) MB and (**c**,**f**) Cr(VI).

The maximum adsorption amount and the difficulty of adsorption could be inferred by isotherm fitting. The experimental temperature was room temperature, specifically, 25 °C. The linear fitting formulas of Langmuir and Freundlich are shown in Equations (4) and (5). The fitting parameters are shown in Table 3. Figure 5a,d show the effect of different initial concentrations on adsorption capacity. The Langmuir adsorption isotherm model describes single-layer adsorption on the surface of the adsorbent; the adsorption only occurs in a certain region, and all the active centers do not have transverse interaction and intermolecular steric hindrance so they are energy equivalent and independent. The Freundlich isotherm is a pure experience, which means that the adsorption heat of each adsorption active site is different, and the adsorbent binds to the active site according to the strength of the binding ability which best describes the adsorption of heterogeneous surfaces. After fitting, as shown in Figure 5b–f, the Langmuir fitting coefficient of the MGPs’ adsorption of MB reached 0.99, which was significantly higher than Freundlich’s fitting coefficient of 0.95, indicating that the MGPs’ adsorption of MB was mainly monolayer adsorption. *K_L_* = 0.06, in the range of 0~1, indicating that the MGPs’ adsorption of MB was favorable and reversible, and the adsorption performance was good. For the fitting results of the MGPs’ adsorption of Cr(VI), the Freundlich fitting coefficient was 0.97, which was much higher than the Langmuir fitting coefficient of 0.88, indicating that the MGPs’ adsorption of Cr(VI) was mainly multi-layer adsorption, and 1/n = 2.5 indicated that the adsorption was difficult [39].

(4) $$\ln q_{e}=\ln K_{F}+\frac{1}{n}\ln C_{e}$$

(5) $$\frac{C_{e}}{q_{e}}=\frac{1}{q_{m}K_{L}}+\frac{C_{e}}{q_{m}}$$

where *q_e_* (mg/g) and *C_e_* (mg/L) are the same as above; 1/n is the intensity of adsorption or affinity; *q_m_* (mg/g) is the maximum sorption amount; *K_F_* (mg/g) and *K_L_* (L/mg) are the Freundlich adsorption constant and Langmuir constant.

### 2.5. Competitive Adsorption

**Figure 6:**
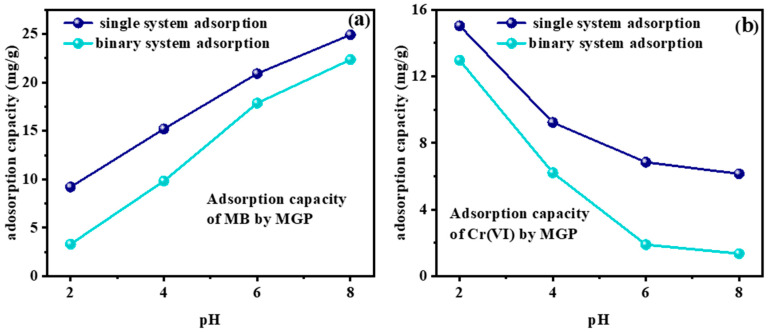
Adsorption of (**a**) MB and (**b**) Cr(VI) by MGP in single and binary systems.

To explore the interactions of MB and Cr(VI) during competitive adsorption in a binary system, various pH values were investigated (Figure 6). The adsorption capacity of MGPs for MB and Cr(VI) in the mixed system was demonstrated with changes in pH. As the pH increased, the adsorption capacity of both MB and Cr(VI) in the binary system decreased compared with that in the single system. When pH = 2, the adsorption capacity of MB by the MGPs decreased from 9.2 mg/g to 3.3 mg/g. The adsorption capacity of Cr(VI) by the MGPs decreased from 15.04 mg/g to 12.96 mg/g, and Cr(VI) was preferentially adsorbed in the binary system, consistent with the experimental results observed under varying pH conditions during the adsorption experiments. Furthermore, when the pH increased and exceeded 6, the adsorption capacity of MB decreased from 20.91 mg/g to 17.86 mg/g, and for Cr(VI) decreased from 6.84 mg/g to 1.89 mg/g, and MB preferentially adsorbed under these conditions.

### 2.6. Desorption Rate and Regeneration

**Figure 7:**
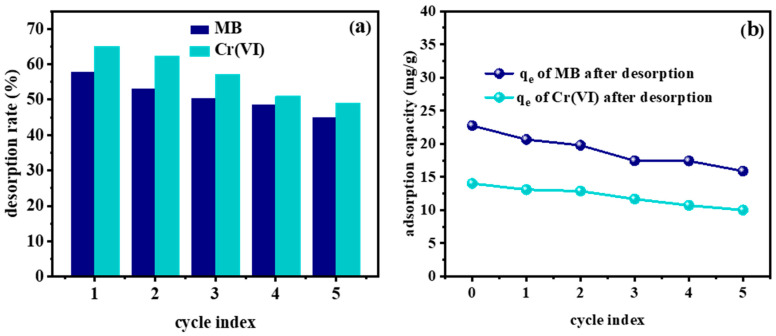
(**a**) Desorption rate and (**b**) adsorption capacity after desorption of MB and Cr(VI).

Figure 7a reflects the desorption rate results of MB and Cr(VI) (100 mg/L; pH = 8 and 2; contact time: 120 min) on the MGPs (0.05 g) after five adsorption–desorption regeneration experiments. It can be seen that the desorption rates of MB and Cr(VI) were still above 45% after five cycles. Furthermore, the desorbed geopolymers also showed a good adsorption effect on MB and Cr(VI), indicating that the molecular skeleton structure of desorbed geopolymers was not destroyed during the desorption process. Figure 7b shows that the adsorption capacity of MB and Cr(VI) could still reach 15.02 mg/g and 10.02 mg/g, respectively, after five cycles, which further proved that the removal effect of the resultant geopolymers for MB was stable and sustained.

### 2.7. Adsorption Mechanism Analysis

**Figure 8:**
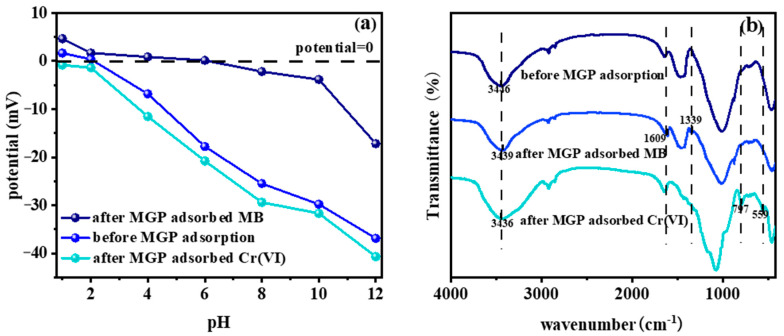
(**a**) Zeta potential and (**b**) FTIR spectra of MGP before and after adsorption.

Zeta potential indicates the mutual repulsion or attraction force between particles. Figure 8a illustrates the zeta potential shifts of the MGPs during adsorption. As the pH rose, the MGPs’ potential decreased. The zero electric point was at pH = 2. Beyond this pH, the geopolymers’ high hydroxide content rendered it negatively charged. The adsorption of MB shifted the potential of the MGPs to the right, raising its zero electric point to pH = 6. This shift occurred because MB ionizes positively charged groups. The sharp potential drop at pH > 10 was due to the strong alkalinity of the solution. In contrast, Cr(VI) adsorption led to a leftward shift in the surface potential of the MGPs, eliminating the zero electric point. This was attributed to potassium dichromate’s ability to ionize negatively charged chromate and hydrogen chromate. The zeta potential changed during Cr(VI) adsorption by the MGPs, following a similar trend to that of unadsorbed MGPs.

As can be seen from the FTIR patterns in Figure 8b, the peak at 3446 cm^−1^ indicates the presence of hydroxyl groups on the surface of the MGPs [43,44,45]. The stretching vibration at 1609 cm^−1^ is caused by the aromatic ring of methylene blue, and the bending vibration of methyl at 1339 cm^−1^ is also evidence of MB adsorption on the MGPs. Potassium dichromate was also successfully adsorbed on MGPs, and the peak at 797 cm^−1^ represents the tensile vibration of Cr-O. The vibration at 559 cm^−1^ indicates Cr(III)-OH, which suggests that part of Cr(VI) was reduced to Cr(III) and adsorbed on the MGPs.

**Figure 9:**
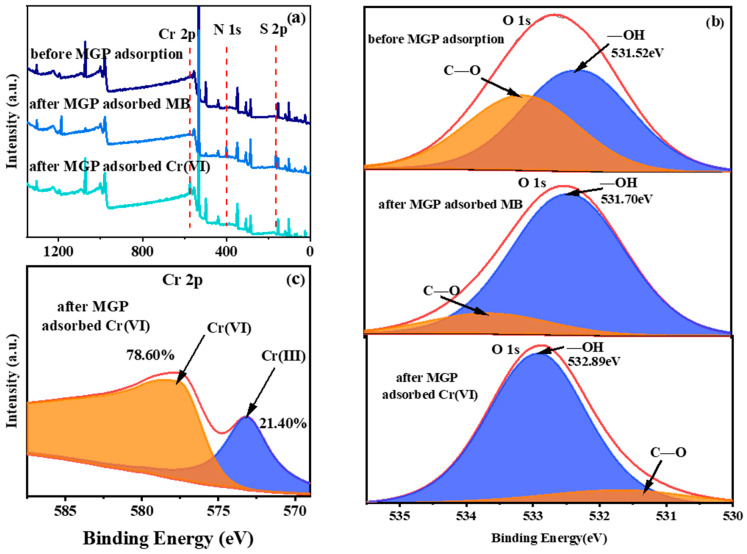
(**a**) Full spectrum and (**b**) O1s of MGP before and after adsorption, (**c**) Cr2p in MGP after Cr(VI) adsorption.

Figure 9a shows the full spectra of XPS before the MGPs’ adsorption, after the MGPs’ adsorption of MB and after the MGPs’ adsorption of Cr(VI). It can be seen that, after the adsorption of MB and Cr(VI), there were Cr peaks, N peaks and S peaks in the spectra that were not present before adsorption, which indicates that the MGPs successfully adsorbed MB and Cr(VI). In Figure 9b, before and after MB and Cr(VI) adsorption, the -OH peak of O1s shifts to the left, indicating that the hydroxyl group in the MGPs reacted with MB and Cr(VI). This is possibly because the N atoms contained in MB connected with the hydroxyl group to form a nitrogen-linked hydroxyl group structure. As shown in Figure 9c, the amount of Cr(III) was 21.4%, while Cr(VI) accounted for 78.6% [38]. A part of hexavalent chromium was adsorbed on the MGPs by reduction to trivalent chromium. It was shown that some reductive substances (hydroxyl group on the surface of MGPs) in MGPs underwent redox reactions with Cr(VI).

**Figure 10:**
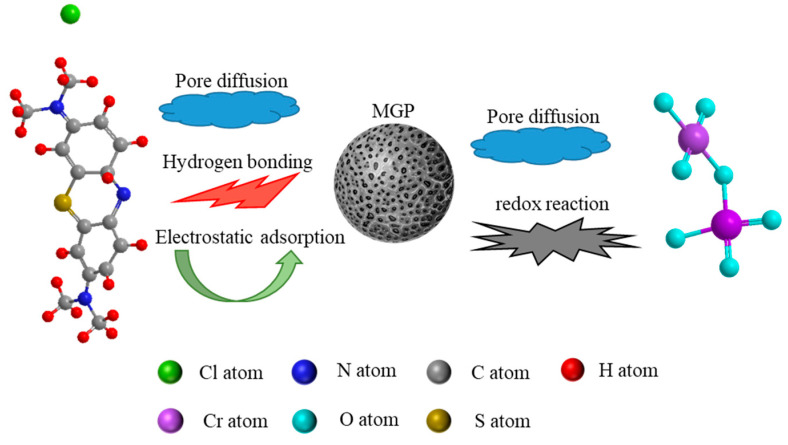
Proposed adsorption mechanism of MB and Cr(VI) on MGP.

The adsorption mechanisms of MGPs for MB and Cr(VI) are shown in Figure 10. During the adsorption process, the rough surface and pore structure of the MGPs provided a large number of adsorption sites for MB molecules and Cr(VI), and MB and Cr(VI) were successfully attached to the MGPs through surface diffusion and pore diffusion. The N atoms on the MB molecules also formed a hydrogen bond with the hydroxyl group contained in the MGPs. At the same time, there was electrostatic adsorption between the negative charge on the MGPs’ surface and the positive group ionized by MB. When Cr(VI) was adsorbed by the MGPs, in addition to pore diffusion, Cr(VI), with its strong oxidation capacity, also reacted with the hydroxyl group in the MGPs.

### 2.8. Comparison of Adsorption Properties of Geopolymer Adsorbents

In order to compare the adsorption performance reported in this article with that reported in other literature, relevant information is presented in Table 4. It can be observed that the majority of polymeric adsorbents are made from MK, which, indeed, exhibits good adsorption performance for pollutants. However, kaolin is a non-renewable resource and incurs higher costs compared to industrial solid waste. Table 4 also indicates that most of the polymeric adsorbents currently target single-pollutant adsorption, and there has been limited research on competitive adsorption. This study, on the other hand, utilized industrial solid waste as a raw material, investigating both single-system adsorption and competitive adsorption of organic and inorganic pollutants while also demonstrating promising recyclability.

## 3. Materials and Method

### 3.1. Materials

**Figure 11:**
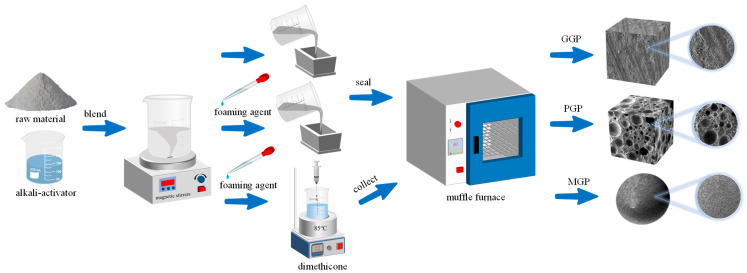
Schematic diagram illustrating the synthesis routes of GGP, PGP and MGP.

CG and BFS were procured from Hebei Province, while FA was procured from Henan Province. Hydrogen peroxide (H_2_O_2_) was purchased from the Aladdin company and contained 30% H_2_O_2_. Table 5 shows the synthesis schemes of the geopolymers: GGPs, PGPs and MGPs. Figure 11 shows the preparation process for the three geopolymers. The alkali activator was prepared 24 h in advance. A certain proportion of solid waste powder was poured into the alkali activator, stirred fully for 5 min and then placed into the mold. GGPs were prepared by pouring method. PGPs were directly foamed, and H_2_O_2_ was added after the slurry was mixed. Then, the polymer paste was molded and cured in an oven at 80 °C for 6 h and then cured at room temperature for 7 days. MGPs were suspended for curing. The prepared slurry was uniformly dropped into a reaction kettle equipped with dimethyl silicone oil at 85 °C and was quickly dispersed into round microspheres under the stirring of a disperser. After filtration and collection, it was cured in an oven at 80 °C for half an hour and then calcined in a muffle furnace at 300 °C for 2 h to remove excess oil.

Before the adsorption experiments, the GGPs, PGPs and MGPs were washed in distilled water until neutral. The initial conditions of MB (or Cr(VI)) adsorption experiment were 25 °C, pH = 6 (or pH = 2), and 50 mg adsorbent was placed in 50 mL MB (or Cr(VI)) solution at a concentration of 100 mg/L (or 50 mg/L) and stirred for 2 h using an 800 r/min magnetic agitator.

The batch adsorption experiments were divided into multiple categories: adsorbent type, adsorbent dosage, initial concentration of MB and Cr(VI) and adsorption pH. The single variable adsorption method was adopted. The removal efficiency and adsorption capacity calculation formulas are presented in Equations (6) and (7), respectively.

(6) $$r=\frac{C_{0}-C_{e}}{C_{0}}\times 100\%$$

where *r* is the removal rate of MB or Cr(VI) at the given time, *C*_0_ (mg/L) and *C_e_* (mg/L) are the initial and given time concentrations of MB or Cr(VI).

(7) $$q_{e}=\frac{\left(C_{0}-C_{e}\right)V}{m}$$

where *q_e_* (mg/g) is the adsorbed amount of MB or Cr(VI) per unit weight of MGP in an equilibrium solution, respectively; *V* (L) is the volume of MB or Cr(VI) solution; *m* (g) is the weight of MGP; *C*_0_ (mg/L) and *C_e_* (mg/L) are initial and equilibrium concentrations.

### 3.2. Characterization

The chemical analysis of CG, FA and BFS was performed by an X-ray fluorescence instrument (Axios Advanced, Almelo, The Netherlands). The structure and composition of CG were tested by the Brucker D8 X-ray diffraction meter (Karlsruhe, Germany). The FTIR spectrometer of Vector-22 Bruker from Germany was employed to analyze the evolution of changes before and after adsorption of geopolymers. The pore sizes of geopolymers were reported by a mercury injection apparatus (AutoPore 9500, Micromeritics Instrument Corporation, Knoxville, TN, USA). The surface structure of the geopolymers was characterized by SEM (Quanta 250, FEI Company, Peabody, MA, USA) at an accelerating voltage of 0.2–30 kV. Samples were sputtered with gold before the SEM measurements. Changes of binding energy before and after adsorption were obtained by X-ray photoelectron spectrometer (ESCALAB 250, Thermo Fisher Scientific, Waltham, MA, USA) at 0.6 eV energy resolution. The electrokinetic properties of the geopolymers were measured by a Zetasizer Nano-ZS90 instrument (Malvern Panalytical, Malvern, UK) with electrophoretic light-scattering technology.

## 4. Conclusions

In this work, three kinds of geopolymer, GGPs, PGPs and MGPs, were successfully prepared from industrial solid waste using the pouring method, direct foaming method and suspension curing method, respectively. These have the potential to be green and low-cost adsorbents for industrial wastewater.

The specific surface area and porosity of the PGPs and MGPs were higher than those of the GGPs, and the adsorption effects of MB and Cr(VI) were significantly higher than those of GGPs and PGPs. MGPs exhibited advantages such as strong adsorption capacity, rapid preparation, no need for crushing and easy recovery.

The MGPs could adsorb both anionic pollutant MB and cationic pollutant Cr(VI). The adsorption of MB by the MGPs was more consistent with the pseudo-second-order kinetic model and Langmuir isotherm model, while the adsorption of Cr(VI) aligned more closely with the pseudo-first-order kinetic model and Freundlich isotherm model. The adsorption capacity reached 26.8 mg/g and 27.12 mg/g, respectively, and the removal rates were 94.56% and 79.46%, respectively. The competitive adsorption experiments revealed that MB and Cr(VI) inhibited each other during the adsorption process. The adsorption mechanisms of MB were primarily pore diffusion, formation of hydrogen bonds and electrostatic adsorption, whereas the adsorption mechanism of Cr(VI) was mainly pore diffusion, accompanied by the occurrence of redox reactions. Through five cycles of experiments, it was demonstrated that the MGPs exhibited excellent recyclability performance.

## Figures and Tables

**Table 1:** Elemental composition of CG, FA and BFS.

Components(%)	SiO_2_	Al_2_O_3_	CaO	TiO_2_	K_2_O
CG	63.99	25.06	0.56	1.30	3.72
FA	53.08	28.64	0.04	2.39	1.58
BFS	30.57	15.09	38.55	1.60	0.37

**Table 2:** Adsorption kinetic fitting parameters of MGP.

Pseudo-First-Order Kinetic			Pseudo-Second-Order Kinetic			Intraparticle Diffusion					
***K*_1_**	***q_e_***	**R^2^**	***K*_2_**	***q_e_***	**R^2^**	***K*_i1_**	***P*_1_**	**R_1_^2^**	***K*_i2_**	***P*_2_**	**R_2_^2^**
0.003	30.44	0.99	0.00024	46.19	0.99	1.24	9.98	0.99	0.36	31.04324	0.9998
0.020	14.44	0.9998	0.00124	18.44	0.996	1.4	−0.56	0.98	0.29	10.92	0.87

**Table 3:** Isotherm fitting parameters of MGP.

Langmuir Isotherm			Freundlich Isotherm		
***q_m_***	***K_L_***	**R^2^**	***K_F_***	***n***	**R^2^**
33.06	0.06	0.99	5.45	2.8	0.95
29.64	0.08	0.88	0.01	0.4	0.97

**Table 4:** Comparison of adsorption properties of various geopolymer adsorbents for pollutants.

Raw Materials	Shape	Pollutants	Maximum Adsorption Capacity	Reusability	References
CG, FA and BFS	Microsphere	MB and Cr(VI), single and binary	26.8 mg/g 27.12 mg/g	Five cycles	This work
MK	Microsphere	MB, single	4.2 mg/g	Not given	[46]
Biomass FA	Block	MB, single	15.4 mg/g	Five cycles	[47]
Biomass FA	Microsphere	MB, single	30.1 mg/g	One cycle	[37]
MK (organic, modified)	Powder	Cu(II) and Cr(VI), single and binary	108.2 mg/g 95.3 mg/g	Not given	[39]
MK	Powder	Pb(II), Cd(II), Cu(II) and Cr(III), single	100 mg/g 75.74 mg/g 54.54 mg/g 10.15 mg/g	Not given	[48]

**Table 5:** Raw material ratios for preparation of GGP, PGP and MGP.

Samples	Proportion						
	CG/g	FA/g	BFS/g	NaOH/g	Na_2_SiO_3_ 9H_2_O/g	H_2_O/g	H_2_O_2_/mL
GGP	50	50	—	5.81	40	17.2	—
PGP	50	50	—	5.81	40	17.2	1.0
MGP	25	25	50	5.81	40	17.2	1.0

## Data Availability

Data are contained within the article.
